# Chiral topological light for detection of robust enantiosensitive observables

**DOI:** 10.1038/s41566-024-01499-8

**Published:** 2024-08-13

**Authors:** Nicola Mayer, David Ayuso, Piero Decleva, Margarita Khokhlova, Emilio Pisanty, Misha Ivanov, Olga Smirnova

**Affiliations:** 1 Max-Born-Institut, Berlin, Germany; 2 Attosecond Quantum Physics Laboratory, Department of Physics, King’s College London, London, UK; 3 Department of Physics, Imperial College London, London, UK; 4 School of Physical and Chemical Sciences, Queen Mary University of London, London, UK; 5 CNR IOM and Dipartimento di Scienze Chimiche e Farmaceutiche, Universitá degli Studi di Trieste, Trieste, Italy; 6 Department of Physics, Humboldt Universität zu Berlin, Berlin, Germany; 7 Technion—Israel Institute of Technology, Haifa, Israel; 8 Technische Universität Berlin, Berlin, Germany

**Keywords:** Nonlinear optics, High-harmonic generation, Circular dichroism

## Abstract

The topological response of matter to electromagnetic fields is a highly demanded property in materials design and metrology due to its robustness against noise and decoherence, stimulating recent advances in ultrafast photonics. Embedding topological properties into the enantiosensitive optical response of chiral molecules could therefore enhance the efficiency and robustness of chiral optical discrimination. Here we achieve such a topological embedding by introducing the concept of chiral topological light—a light beam which displays chirality locally, with an azimuthal distribution of its handedness described globally by a topological charge. The topological charge is mapped onto the azimuthal intensity modulation of the non-linear optical response, where enantiosensitivity is encoded into its spatial rotation. The spatial rotation is robust against intensity fluctuations and imperfect local polarization states of the driving field. Our theoretical results show that chiral topological light enables detection of percentage-level enantiomeric excesses in randomly oriented mixtures of chiral molecules, opening a way to new, extremely sensitive and robust chiro-optical spectroscopies with attosecond time resolution.

## Abstract (web summary)

The concept of chiral topological light—a polychromatic light with chiral closed 3D polarization trajectories, space-varying with the azimuthal angle—is introduced and used for efficient sensing in chiral molecules, showcasing an example of successful application of topological concepts in optics.

## Main

The topological properties of the electronic response to electromagnetic fields in solid state systems, as well as in photonic structures, are being actively exploited to obtain robust observables, such as, for example, edge currents protected from material imperfections in topological insulators^1^ or topologically protected light propagation pathways in their photonic analogues^2,3^. A similar robustness in the enantiosensitive optical response of gases or liquids of chiral molecules is strongly desired for analytical purposes, but is currently missing. Although the first ideas connecting topological and chiral properties of electronic responses^4^ or microwave signals in molecular gases^5,6^ are starting to emerge, they do not map onto the optical response, which encodes the ultrafast chiral electronic dynamics^7^.

Topologically non-trivial optical signals can be achieved by using vortex beams, which carry orbital angular momentum (OAM). They are characterized by an integer topological charge representing the number of helical revolutions of light’s wavefront in space within one wavelength^8^. Recent work established the chirality of vortex light in the linear regime^9,10^, and exploited and manipulated ultrafast non-linear optical responses to vortex beams in atoms^11,12^, leading to the discovery of new synthetic topologies^13^, as well as in chiral molecules^14,15^, for which vortex light has also been successfully used for chiral detection in the hard X-ray region^16,17^. However, its natural enantiosensitivity in the optical domain is weak: the spatial scale of optical vortex beams is many orders of magnitude larger than the size of a molecule, making it difficult for the molecule to sense global field structures.

This limitation can be overcome by encoding chirality of the optical field in time rather than in space. This means that locally, at a fixed point in space, the electric field vector of the electromagnetic wave draws a chiral three-dimensional Lissajous figure during one laser cycle. Fields with such chiral Lissajous figures, referred to as synthetic chiral light^18^, employ only electric-dipole transitions to drive nonlinear enantiosensitive signals. They have been devised^19^, applied in the microwave region^20^ and extended to the optical domain^18^. The handedness of this light can be controlled with the phase delay between its frequency components, both locally—at every point in space—and globally in the interaction region^21^.

Here we introduce the concept of chiral topological light, which takes advantage of the global topological structure of vortex light and the high enantiosensitivity of synthetic chiral light^18^, embedding robust topological properties into the highly enantiosensitive ultrafast optical response.

Our key idea is to imprint the topological properties of the vortex beam onto the synthetic chiral light. Locally, the handedness of this light is characterized by the chiral correlation function *h* (ref. ^18^). We therefore aim to imprint the topological charge of the vortex beam on the azimuthal phase of *h*: $\arg [h(\theta )]=C\theta +{\phi }_{\rm{L}}$. Here *θ* is the azimuthal angle, *C* is the topological charge and *ϕ*_L_ is the local enantiosensitive phase of the complex-valued correlation function *h*.

We now show that the intensity of the nonlinear optical emission of a chiral molecular medium triggered by such light depends on both chiral and topological phases of *h* as well as the enantiosensitive phase *ϕ*_M_ introduced by the molecular medium: $I(\theta )\propto \cos ({\phi }_{\rm{M}}-{\phi }_{\rm{L}}+C\theta )$. We find that the azimuthal intensity profile is patterned in a topologically robust and molecule-specific way, leading to a large enantiosensitive offset Δ*θ* = π/*C* between the intensity maxima (or minima) in opposite enantiomers. Furthermore, we find that the topologically controlled angular offset is robust with respect to imperfections of light polarization and intensity fluctuations, and persists for very small amounts of enantiomeric excess (e.e.). It can therefore be used to probe chirality in dilute mixtures.

**Fig. 1:**
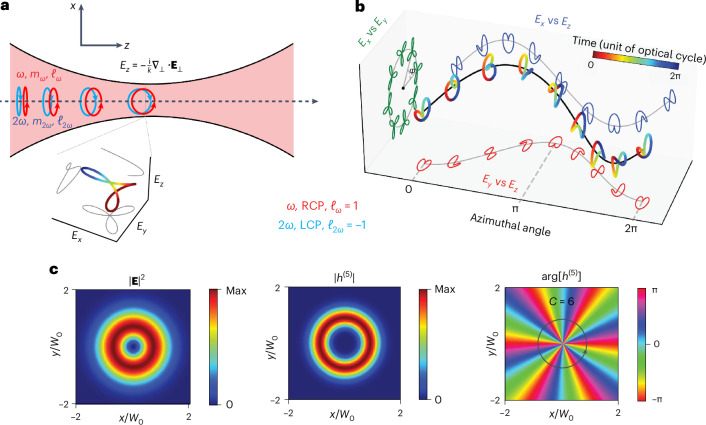
Chiral topological light. The concept of chiral vortex light for bicircular counter-rotating *σ*_*ω*_ = −*σ*_2*ω*_ = 1 beams carrying OAM *ℓ*_*ω*_ = −*ℓ*_2*ω*_ = 1. **a**, Tight focusing of bicircular-counter-rotating Gaussian beams induces a longitudinal field, resulting in a synthetic chiral field whose polarization vector draws a chiral Lissajous curve over one laser cycle (inset). **b**, Evolution of the chiral Lissajous curves with respect to the azimuthal angle *θ* at a given radial position $\rho =\sqrt{{x}^{2}+{y}^{2}}$ at *z* = 0 for a chiral vortex with *ℓ*_*ω*_ = −*ℓ*_2*ω*_ = 1. **c**, Slices through the electric field distribution at *z* = 0. The figures show the total intensity of the electric field ∣**E**∣^2^, the absolute value of the chiral correlation function ∣*h*^(5)^∣ and its phase distribution $\arg \left[{h}^{(5)}\right]$. The phase distribution of *h*^(5)^ describes the spatial distribution of the handedness of light and is characterized by a topological charge *C* = 6. The *x* and *y* coordinates are scaled to the waist of the beams (*W*_0_) at the focus.

To demonstrate these ideas, we focus on a specific realization of chiral topological light. It involves two Laguerre-Gaussian beams with counter-rotating circular polarizations, propagating along the *z*-axis with frequencies *ω* and 2*ω* and OAMs *ℓ*_*ω*_ and *ℓ*_2*ω*_ (see Methods). Near the focus the field develops a longitudinal component given by *E*_*z*_ = −(*i*/*k*)∇_⊥_ ⋅ **E**_⊥_ in the first post-paraxial approximation^22^ (see Fig. 1a), taking the light polarization vector out of the (*x*, *y*) plane—a prerequisite for creating synthetic chiral light.

As a result, the Lissajous figure drawn by the polarization vector in one point in space over a laser cycle becomes chiral (see inset in Fig. 1a). Its handedness is controlled by the two-colour phase *ϕ*_2*ω*,*ω*_ = 2*ϕ*_*ω*_ − *ϕ*_2*ω*_, which depends on the azimuthal coordinate, forming a chiral vortex with the topological charge (see Methods):

1 $$C=2{\ell }_{\omega }-{\ell }_{2\omega }+2{\sigma }_{\omega }-{\sigma }_{2\omega }.$$

Here *σ*_*rω*_ indicates either right (*σ*_*rω*_ = 1) or left (*σ*_*rω*_ = −1) circular polarization. The Lissajous curve drawn by the polarization vector of the electric field over one laser cycle changes with the azimuthal angle, switching handedness 2∣*C*∣ times as the azimuthal angle cycles over one revolution (Fig. 1b). Thus, the superposition of two tightly focused OAM beams at commensurate frequencies gives rise to a chiral vortex, that is, a vortex beam displaying chirality locally at each given point, with an azimuthally varying handedness characterized by an integer topological charge *C*.

Figure 1c visualizes the chiral vortex by displaying the beam total intensity ∣**E**(*x*, *y*)∣^2^, the absolute value ∣*h*^(5)^(*x*, *y*)∣ and the phase $\arg [{h}^{(5)}(x,y)]$ of the chiral correlation function for OAM $({\ell }_{\omega },{\ell }_{2\omega })=\left(1,-1\right)$ and spin-angular momentum (SAM) (*σ*_*ω*_, *σ*_2*ω*_) = (1, −1). Both the chirality and the total intensity maximize along rings (see Fig. 1c), typical for vortex beams, whereas the topological charge *C* = 6 characterizes the azimuthal phase distribution of the light’s handedness quantified by the chiral correlation function.

The chiral topological charge *C* is highly tunable thanks to its dependence on the OAM of the two beams, which can take any integer value from −*∞* to *∞*, enabling chiral vortices with arbitrarily high (or low) chiral topological charges. By controlling the OAM of the beams, we can thus create chiral vortex beams with controlled properties. If *C* = 0, then the chiral vortex has the same local handedness everywhere in space. Otherwise, the field’s handedness displays a non-trivial spatial structure, which is characterized by *C*.

**Fig. 2:**
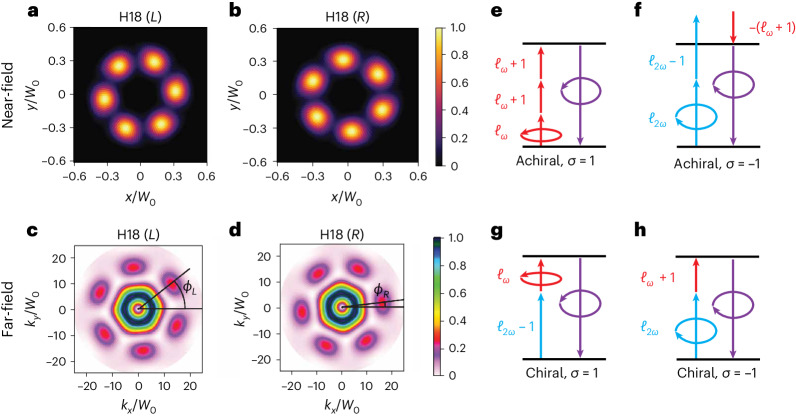
Enantiosensitive high-harmonic spectroscopy using chiral topological light with topological charge *C* = 6. **a**,**b**, The near-field spatial profile of H18 for *L*-fenchone (**a**) and *R*-fenchone (**b**). The *x*- and *y*-axes are given in units of the field waist at the focus *W*_0_. **c**,**d**, The corresponding far-field spatial profiles for the two enantiomers are shown in **b** for *L*-fenchone and **d** for *R*-fenchone. Here *k*_*x*_ and *k*_*y*_ are given in units of the reciprocal waist of the field at the focus (1/*W*_0_). All profiles are normalized to their maximum value, which is the same for opposite enantiomers. The angles in the far-field picture indicate the position of the first peak in the outer ring of the profile, where we set the zero angle along the positive *k*_*x*_ direction. For *C* = 6, we have that *ϕ*_L_ = *ϕ*_R_ + π/3. **e**–**h**, Multiphoton diagrams describing the generation of 3*N* high-harmonic orders in a chiral molecule driven by chiral topological light. Photons carrying SAM *σ* = ±1 are indicated with co- or counter-clockwise arrows, whereas longitudinally polarized photons are simple arrows. The *ℓ*_*rω*_ term corresponds to the OAM carried by each photon. **e**,**f**, The achiral channels (odd number of photons), where **e** corresponds to a final SAM of *σ* = 1 and **f** to *σ* = −1. **g**,**h**, The chiral channels (even number of photons), for a final SAM of *σ* = 1 (**g**) or *σ* = −1 (**h**). **e**–**h**, A final SAM of the 3*N* order of *σ* = 1 (**e**,**g**) or *σ* = −1 (**f**,**h**).

We have modelled the highly nonlinear response of randomly oriented chiral molecules to this realization of chiral topological light depicted in Fig. 1 using a density-functional-theory (DFT)-based *S*-matrix approach (see Methods). Figure 2a,b shows the near-field intensity of harmonic 18 (H18) generated in *R*- and *L*-fenchone for a fundamental frequency of *ω* = 0.044 a.u. (1,033 nm), peak intensity of *I*_0_ = 5 × 10^14^ W cm^–^^2^ and beam waist of *W*_0_ = 2.5 μm at the jet position *z* = 0.

The azimuthal distribution of the near-field intensity records both the topology of the driving laser field and the handedness of the medium. This azimuthal distribution results from the interference between chiral and achiral multiphoton pathways. The maxima occur at angles $\theta =\left[2\uppi n+({\phi }_{\rm{L}}-{\phi }_{\rm{M}})\right]/C$, where the two pathways interfere constructively. The angular position of the peaks is therefore enantiosensitive: swapping the molecular enantiomer leads to a π shift in the molecular phase *ϕ*_M_ → *ϕ*_M_ + π, shifting the minima and maxima of the intensity pattern by π/*C*. The number of peaks is controlled by the topological charge ∣*C*∣ = 6. Importantly, the same topological structure is preserved in the far-field response (Fig. 2c,d). In the multiphoton picture of high-harmonic generation (HHG), the enantiosensitive topological structure arises due to the interference between achiral and chiral channels^18^, which we depict in Fig. 2e–h. Taking into account both the SAM *σ* and OAM *ℓ* carried by each photon, it is easy to see that the difference in net OAM transferred to the harmonic orders in chiral and achiral channels corresponds to the topological charge *C* (see Methods for a detailed description).

**Fig. 3:**
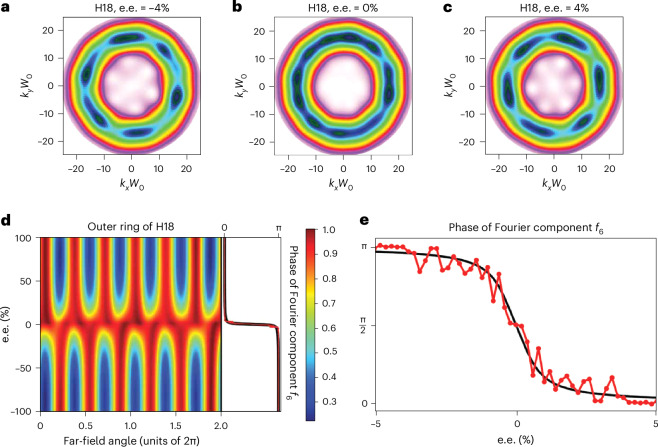
Dependence of the signal on the enantiomeric excess. **a**–**c**, The far-field spatial profiles of H18 for e.e. = (*N*_R_ − *N*_L_)/(*N*_R_ + *N*_L_) –4% (**a**), 0% (**b**) and 4% (**c**), where a positive e.e. corresponds to a larger concentration of *R*-fenchone in the sample. **d**, Angle-resolved, radially integrated far-field signal of the outer ring of the spatial profile (∣*kW*_0_∣ > 10) as a function of e.e. The black line on the right shows the phase of the Fourier component of the spatial profile oscillating at frequency *ℓ* = 6 as a function of e.e. The overlapping red line shows the result accounting for intensity fluctuations. The π jump at e.e. = 0% indicates the enantiosensitive rotation of the spatial profile. **e**, The phase of the Fourier component is shown by a black solid line. The red line with circles shows the phase obtained, including the intensity fluctuations for e.e. between –5% and 5%.

Encoding the topological charge *C* into the molecular response and extracting the enantiosensitive offset angle, controlled by *C*, allows us to measure the e.e. = (*N*_R_ − *N*_L_)/(*N*_R_ + *N*_L_) in macroscopic mixtures of left- and right-handed molecules with concentrations *N*_L_ and *N*_R_, respectively. Even for very small e.e. values, we observe the appearance of the *C*-fold structure in the inner and outer rings, as well as the corresponding enantiosensitive rotation of the spatial profile (Fig. 3a,c). For e.e. = 0% (Fig. 3b), chiral channels are suppressed, and a topologically different 2*C*-fold structure is observed as a result of the interference between the two strongest open achiral channels that lead to H18, and allowed by the selection rules (Fig. 2e,f; see Methods and Supplementary Information for more details).

The enantiosensitive rotation of the *C*-fold structure in the outer ring is apparent in the angle-resolved, radially integrated signal (Fig. 3d). It manifests in the abrupt switching of the azimuthal angle, which maximizes the signal as one changes the e.e. from positive to negative. The enantiosensitive rotation can be easily separated by performing a Fourier analysis of the signal with respect to the azimuthal angle as a function of the e.e. The solid black line in Fig. 3d shows the phase of the Fourier component *f*_6_ oscillating at the *C* = 6 frequency of the outer ring signal as a function of e.e. A clear π phase jump is observed at e.e. = 0%, indicating the switch in the handedness of the mixture. The sharpness of this jump (Fig. 3e) characterizes the accuracy of resolving left- and right-handed molecules in mixtures with vanishingly small e.e.

We now show that the enantiosensitive signal is robust with respect to imperfections in the laser beams. First, we include noise in our simulations (see Methods) via 2% intensity fluctuations of the driving fields. The red line in Fig. 3d,e shows the phase of the Fourier component *f*_6_ when intensity fluctuations are included. It is clear that the sharp π shift of the phase is robust against noise (see Fig. 3e). It allows us to distinguish positive and negative e.e. with high fidelity, on the scale of ~0.1%, demonstrating topological robustness of the enantiosensitive signal. The topological structure is imprinted via azimuthal interference of chiral and achiral responses and survives as long as the two-colour phase remains stable, which is routinely achieved in two-colour experiments with extremely high accuracy (see, for example, ref. ^23^). We thus expect robust encoding of topological information in the molecular gas and robust read-out of the chiral topological signal.

Figure 3 is the first key result of our work: our method is sensitive to very small values of e.e. in nearly equal mixtures of left- and right-handed molecules. This sensitivity—at well below 1%—rivals or even exceeds the golden standard achieved in photo-electron spectroscopy^24,25^ via the new generation of chiral-sensitive methods that are based on electric dipole interactions^26–30^.

Typical experimental imperfections are related to the imperfect circularity (SAM) and imperfect OAM contents of the light beams. We show below that although such imperfections affect the topological charge, the concept of enantiosensitive rotation of the non-linear response remains valid.

**Fig. 4:**
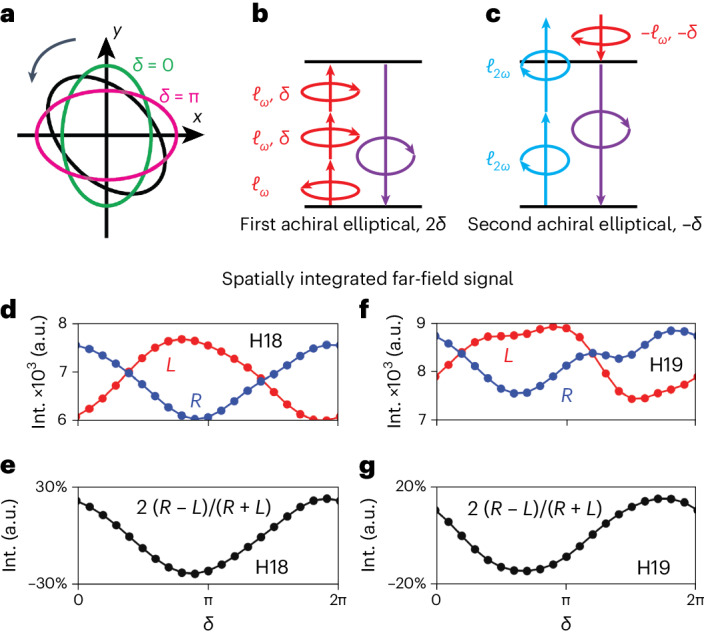
Fourier analysis to recover the enantiosensitive rotation of the spatial profile in the case of elliptical fields. **a**, Ellipse of an elliptical field and its orientation in the (*x*, *y*) plane with respect to the phase delay *δ* between the counter-rotating components. **b**,**c**, Multiphoton diagrams of the new achiral channels contributing to the 3*N* harmonic orders for chiral topological light with an elliptical *ω* field, where **b** corresponds to a final phase delay dependence of 2*δ* and **c** to a final phase delay dependence of −*δ.* The co- and counter-clockwise arrows indicate the SAM *σ*, whereas *ℓ*_*ω*_ corresponds to the OAM; *δ* is the phase delay *δ*. **d**–**g**, Spatially integrated far-field signal for H18 (**d**,**e**) and H19 (**f**,**g**) as a function of the phase delay *δ*. The red (blue) dotted lines in **d** and **f** corresponds to the signal *S*_L_ (*S*_R_) from *L*(*R*)-fenchone, whereas the black dotted lines in **e** and **g** correspond to the chiral dichroism signal 2(*S*_R_ − *S*_L_)/(*S*_R_ + *S*_L_). Int., intensity.

Consider chiral topological light created by elliptically polarized drivers with imperfect circularity (see the Supplementary Information for imperfections in the OAM content). To understand its effect on our observables, we express the elliptical field in terms of two counter-rotating circularly polarized components: ${{{\bf{E}}}}(\omega )=[(1+\epsilon ){{{{\bf{E}}}}}_{+}(\omega )+(1-\epsilon )\exp ({{{\rm{i}}}}\delta\,){{{{\bf{E}}}}}_{-}(\omega )]/\sqrt{2(1+{\epsilon }^{2})}$. Here *δ* is the phase delay between the components, which corresponds to the orientation of the resulting elliptical polarization and can be well controlled in the experiment^24^, and ∣*ϵ*∣ ≤ 1 is the ellipticity, which is difficult to control with few-percent accuracy. Note that *δ* = 0, π correspond to elliptical light ‘squeezed’ along the *x*- and *y*-axes, respectively (see Fig. 4a), and that both *δ* and *ϵ* can be related to standard Stokes parameters^31^.

The appearance of the additional counter-rotating component in the elliptical beam leads to two inter-related consequences: (1) the change of the topological structure of the harmonic emission due to the presence of new SAM components in the beams (see equation (1)), which leads to admixture of emission with topological charge *C* = –2; and (2) the appearance of two strong multiphoton pathways contributing to the achiral harmonic signal and effectively masking a weaker chiral signal driven by the longitudinal polarization. The new achiral multiphoton channels that arise due to imperfect circularity of the pulse are shown in Fig. 4b,c (see Methods for further details). The contribution of different topological charges and different multiphoton pathways are disentangled by realizing an analogue of the lock-in method: we use the dependence of the signal on *δ*, that is, on the orientation angle of the polarization ellipse. This dependence is different for the different nonlinear optical diagrams carrying different topological charges. Hence, rotating the polarization ellipse, that is, changing *δ* and Fourier transforming the signal with respect to *δ* allows us to decouple the different contributions to the signal according to their topological charges and consequently amplify the chiral signal.

An additional benefit of using elliptical drivers is the generation of globally chiral light with non-zero topological charge. This leads to total (that is, integrated over the spatial profile) enantiosensitive intensity across all harmonic orders, providing an opportunity to harvest not only the relatively weak 3*N* harmonics, but also the naturally intense 3*N* + 1 harmonics. Figure 4d–g shows the total far-field intensity for H18 (Fig. 4d,e) and H19 (Fig. 4f,g) as a function of the phase delay *δ* between the counter-rotating components of the *ω* field for both *R*- (*S*_R_, blue dotted line) and *L*-fenchone (*S*_L_, red dotted line), as well as the chiral dichroism 2(*S*_R_ − *S*_L_)/(*S*_R_ + *S*_L_) (black dotted line), for globally chiral topological light with a fundamental beam ellipticity of *ϵ*_*ω*_ = 0.9. The other parameters are kept as above. The strength of the far-field signal changes as one rotates the ellipse of the *ω* field, whereas the chiral dichroism in the signal intensity is maximized at around 20% and 30% for harmonics 19 and 18, respectively.

**Fig. 5:**
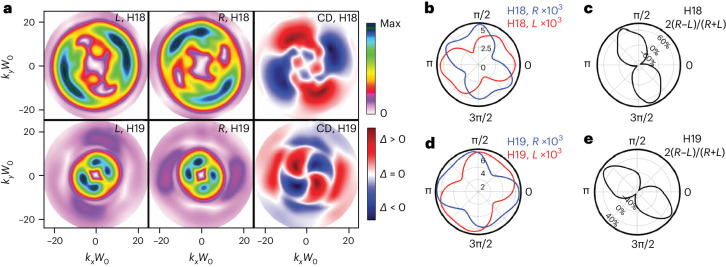
Results of the Fourier analysis for elliptical fields. **a**, Far-field spatial profiles for H18 and H19 obtained after Fourier transform with respect to the phase delay *δ* at the $\tilde{\delta }=1$ Fourier component for H18 (top) and H19 (bottom). The left (centre) column shows the results for *L*(*R*)-fenchone, whereas the right column shows the difference signal S_*R*_ − *S*_*L*_. **b**–**e**, Radially integrated signal as a function of the azimuthal angle of the far-field spatial profiles for H18 (**b**,**c**) and H19 (**d**,**e**). The solid red (blue) lines in **b** and **d** correspond to *L*(*R*)-fenchone, whereas the black lines in **c** and **e** show the chiral dichroism signal 2(*S*_R_ − *S*_L_)/(*S*_R_ + *S*_L_).

Fourier-transforming the far-field intensity profile with respect to the phase delay *δ* separates the contributions of different pathways, because they experience different modulation with *δ*. The two achiral pathways interfere in the third Fourier component ($\tilde{\delta }=3$; see Fig. 4b,c) with respect to *δ*, whereas the dominant contribution between the chiral and achiral pathways corresponds to the first Fourier component ($\tilde{\delta }=1$). Figure 5a shows the far-field spatial profiles for $\tilde{\delta }=1$ for both enantiomers and both H18 and H19, as well as the their difference, whereas the polar plots in Fig. 5b–e show the radially integrated signals and chiral dichroism. We see that the Fourier filtering recovers the enantiosensitive rotation, although the dominant topological charge is now *C* = 2 (equation (1) for *σ*_*ω*_ = *σ*_2*ω*_ = −1).

Figure 5 is the second key result of our work. It demonstrates a robust route to decomposing the contributions to the overall chiral optical signal, originating from interfering pathways encoding different topological charge. The decomposition relies on straightforward Fourier analysis of the far-field image. Given the ability to precisely control the orientation of the polarization ellipse of the incident infrared light, chiral topological light generated by such infrared drivers stands out as a robust probe of molecular chirality, capable of inducing strongly enantiosensitive total intensity signals as well as giant rotations of intense spectral features.

The concept of chiral topological light introduced here is not limited to vortex beams: other members of the larger family of structured light beams^32–34^ can be used to create locally and globally chiral topological light. We envision using tightly focused radially polarized beams, which are known to posses strong longitudinal components^35^, central to the concept of local chirality. Skyrmionic beams^36,37^ could also be used, for example to induce topological distributions with radially dependent topological charges. From the perspective of structured light^32–34,38^ the temporally chiral vortex introduced here represents a new kind of polarization singularity, which could be analysed by extending the current framework from monochromatic 3D fields^39,40^ to polychromatic 3D fields^13,41,42^.

Our method is not limited to high harmonics. Its extension to low-order parametric processes such as chiral sum-frequency generation^43^ has potential for non-destructive enantiosensitive imaging in the ultraviolet region and for exploiting intrinsically low-order nonlinearities for enantiosensitive detection in the X-ray domain^16,17^.

## Methods

### Spatial structure of vortex beams creating chiral topological light

We use two Laguerre-Gaussian beams with counter-rotating circular polarizations, propagating along the *z*-axis with frequencies *ω* and 2*ω*, and OAMs *ℓ*_*ω*_ and *ℓ*_2*ω*_. We set the radial indices to *p*_*ω*_ = *p*_2*ω*_ = 0. The generalization to the case of non-zero radial index is straightforward. At the focal plane of the beams *z* = 0, the Cartesian components of the fields in the transversal plane (*x*, *y*) are

2 $$\begin{array}{rcl}{{{{\bf{E}}}}}_{\pm ,r\omega }^{\perp }&=&{{{{\mathcal{E}}}}}_{r\omega }{\rm{e}}^{-\frac{{\rho }^{2}}{{W}_{0}^{2}}}{\left(\frac{\sqrt{2}\rho }{{W}_{0}}\right)}^{| {\ell }_{r\omega }| }{\rm{e}}^{{{{\rm{i}}}}{\ell }_{r\omega }\theta }{\rm{e}}^{{{{\rm{i}}}}{\phi }_{r\omega }}\\ &&\times \frac{({{{{\bf{e}}}}}_{x}-{{{\rm{i}}}}{\sigma }_{r\omega }{{{{\bf{e}}}}}_{y})}{\sqrt{2}},\end{array}$$

where ${{{{\mathcal{E}}}}}_{r\omega }$ is the field strength; *W*_0_ is the beam waist; *ϕ*_*rω*_ is the carrier-envelope phase; $\rho =\sqrt{{x}^{2}+{y}^{2}}$ and $\theta =\arctan (\,y/x)$ are the radial and azimuthal coordinates, respectively; and *σ*_*rω*_ indicates right (*σ*_*rω*_ = 1) or left (*σ*_*rω*_ = −1) circular polarization. Near the focus, this field develops a longitudinal component along the *z*-axis given by *E*_*z*_ = −(*i*/*k*)∇_⊥_ ⋅ **E**_⊥_ in the first post-paraxial approximation^22^:

3 $$\begin{array}{rcl}{{{{\bf{E}}}}}_{\pm ,r\omega }^{z}&=&-\frac{{{{\rm{i}}}}{{{{\mathcal{E}}}}}_{r\omega }}{\sqrt{2}{k}_{r\omega }}{\rm{e}}^{-\frac{{\rho }^{2}}{{W}_{0}^{2}}}{\left(\frac{\sqrt{2}}{{W}_{0}}\right)}^{| {\ell }_{r\omega }| }{\rho }^{| {\ell }_{r\omega }| -1}\\ &&\times {\rm{e}}^{{{{\rm{i}}}}({l}_{r\omega }+{\sigma }_{r\omega })\theta }\left(| {\ell }_{r\omega }| -{\sigma }_{r\omega }{\ell }_{r\omega }-\frac{2{\rho }^{2}}{{W}_{0}^{2}}\right){{{{\bf{e}}}}}_{z}.\end{array}$$

The total bichromatic electric field **E**(*x*, *y*) = **E**_±,*ω*_ + **E**_±,2*ω*_, which combines the longitudinal and transverse field components for each colour ${{{{\bf{E}}}}}_{\pm ,r\omega }={{{{\bf{E}}}}}_{\pm ,r\omega }^{\perp }+{{{{\bf{E}}}}}_{\pm ,r\omega }^{z}$ (*r* = 1, 2), is an example of a synthetic chiral light^18^.

### Chiral correlation function

We report here the analytical expression^18^ for the chiral correlation function ${h}^{(5)}(-2\omega ,-\omega ,\omega ,\omega ,\omega )={{{{\bf{E}}}}}^{* }(2\omega )\cdot \left[{{{{\bf{E}}}}}^{* }(\omega )\times {{{\bf{E}}}}(\omega )\right]\left({{{\bf{E}}}}(\omega )\cdot {{{\bf{E}}}}(\omega )\right)$ for the general case of two OAM-carrying beams with frequencies *ω* and 2*ω*, SAMs *σ*_*ω*_ and *σ*_2*ω*_, and OAMs *ℓ*_*ω*_ and *ℓ*_2*ω*_.

4 $$\begin{array}{l}{h}^{(5)}(\,\rho ,\theta )=-\frac{{{{{\mathcal{E}}}}}_{2\omega }{{{{\mathcal{E}}}}}_{\omega }^{4}}{\sqrt{2}2{k}_{\omega }^{2}}{\rm{e}}^{-5\frac{{\rho }^{2}}{{W}_{0}^{2}}}{\left(\frac{\sqrt{2}}{{W}_{0}}\right)}^{4| {\ell }_{\omega }| +| {\ell }_{2\omega }| }\\ {\rho }^{4| {\ell }_{\omega }| +| {\ell }_{2\omega }| -3}{\left(| {\ell }_{\omega }| -{\sigma }_{\omega }{\ell }_{\omega }-\frac{2{\rho }^{2}}{{W}_{0}^{2}}\right)}^{2}\\ \left\{\frac{| {\ell }_{\omega }| -{\sigma }_{\omega }{\ell }_{\omega }-2\frac{{\rho }^{2}}{{W}_{0}^{2}}}{2{k}_{\omega }}\left[{\rm{e}}^{i{\sigma }_{\omega }\theta }({\sigma }_{\omega }-{\sigma }_{2\omega })-\right.\right.\\ \left.{\rm{e}}^{-i{\sigma }_{\omega }\theta }({\sigma }_{\omega }+{\sigma }_{2\omega })\right]\\ \left.+\frac{{\sigma }_{2\omega }}{{k}_{2\omega }}\left(| {\ell }_{2\omega }| -{\sigma }_{2\omega }{\ell }_{2\omega }-\frac{2{\rho }^{2}}{{W}_{0}^{2}}\right){\rm{e}}^{-i{\sigma }_{2\omega }\theta }\vphantom{\frac{| {\ell }_{\omega }| -{\sigma }_{\omega }{\ell }_{\omega }-2\frac{{\rho }^{2}}{{W}_{0}^{2}}}{2{k}_{\omega }}}\right\}\\ {\rm{e}}^{i(2{\phi }_{\omega }-{\phi }_{2\omega })}{\rm{e}}^{i(2{\ell }_{\omega }+2{\sigma }_{\omega }-{\ell }_{2\omega })\theta }\end{array}$$

It is easy to verify—in both the counter-rotating *σ*_*ω*_ = −*σ*_2*ω*_ and co-rotating *σ*_*ω*_ = *σ*_2*ω*_ cases—that the azimuthal dependence of the chiral correlation function is given by *Cθ*, where *C* = 2(*ℓ*_*ω*_ + *σ*_*ω*_) − (*ℓ*_2*ω*_ + *σ*_2*ω*_).

### Density-functional-theory-based strong-field approximation simulations in fenchone

We adapt the method from refs. ^18,44,45^ to describe HHG in a chiral molecule subjected to a strong field. The macroscopic dipole moment in an ensemble of randomly oriented molecules arises form the coherent summation of the contributions from all possible molecular orientations

$${{{\bf{D}}}}(N\omega )=\int\,d\Omega \int\,d\beta \,{{{{\bf{D}}}}}_{\Omega \beta }(N\omega ),$$

where *ω* is the fundamental frequency, *N* is the harmonic number and **D**_Ω*β*_ is the harmonic dipole associated with a molecular orientation characterized by the three Euler angles (*θ*, *φ* and *β*), here denoted in terms of the solid angle Ω (that is, $d\Omega=\sin\theta d\theta d\varphi$) and angle *β*. In the strong-field approximation (SFA), the harmonic dipole for a given orientation^18,45^ is given by

5 $$\begin{array}{rcl}{{{{\bf{D}}}}}_{\Omega \beta }(N\omega )&=&{\rm{e}}^{{{{{i}}}}N\omega {t}_{r}^{{\prime} }}{a}_{{{{\rm{rec}}}}}\,{{{\bf{d}}}}({{{{\rm{U}}}}}_{\Omega \beta }{{{\rm{Re}}}}[{{{\bf{k}}}}({t}_{r}^{{\prime} })]){a}_{{{{\rm{prop}}}}}\\ &&{\rm{e}}^{-{{{{i}}}}S({{{{\bf{p}}}}}_{s},{t}_{i},{t}_{r})}\,{a}_{{{{\rm{ion}}}}}\,{\Psi }_{D}({{{{\rm{U}}}}}_{\Omega \beta }{{{\rm{Re}}}}[{{{\bf{k}}}}({t}_{i}^{{\prime} })]),\end{array}$$

where **d**(**k**) is the recombination matrix element in the laboratory frame and **k**(*t*) = **p** + **A**(*t*). Here, U_Ω*β*_ is the rotation matrix that transforms the laboratory frame (**e**_1_, **e**_2_, **e**_3_) to the molecular (**i**_1_, **i**_2_, **i**_3_) frame, with elements U_*ij*_ = 〈**e**_*i*_∣**i**_*j*_〉 for a given orientation. Here, Ψ_*D*_(**k**) = 〈**k**∣Ψ_*D*_〉 is the overlap between the Volkov state with kinetic momentum **k** and the Dyson orbital, where the latter is the overlap between the neutral *N*-electron wavefunction and ionic (*N* − 1) electron wavefunction $\left\vert {\Psi }_{D}\right\rangle =\langle {\Psi }^{N-1}| {\Psi }^{N}\rangle$. The integral over the solid angle $d\Omega =d\alpha d\beta \sin (\beta )$ is performed using the Lebedev quadrature method^46^, whereas the integral over the *β* angle is performed via the trapezoid method. To find the rotation matrix, we first assume that the *x*-axis of the molecular frame points toward a given Lebedev point, and then rotate by an angle *β* around the *x*-axis. For all simulations we use a seventeenth-order Lebedev quadrature (for a total of 110 points) and 40 *β* angles evenly distributed on the [0, 2π] interval.

In the expression for the harmonic dipole **p**, ${t}_{i}={t}_{i}^{{\prime} }+{{{\rm{i}}}}{t}_{i}^{{\prime\prime} }$, ${t}_{r}={t}_{r}^{{\prime} }+{{{\rm{i}}}}{t}_{r}^{{\prime\prime} }$ are the complex momenta, and the times of ionization and recombination that result from the application of the saddle-point method^45^; $S({{{\bf{p}}}},{t}_{i},{t}_{r})=\frac{1}{2}\int_{{t}_{i}}^{{t}_{r}}d{t}^{{\prime} }\,{\left[{{{\bf{p}}}}+{{{\bf{A}}}}({t}^{{\prime} })\right]}^{2}+{I}_{p}({t}_{r}-{t}_{i})$ is the action from the (complex) times of ionization and recombination. The terms associated with the saddle-point method on (*t*_*i*_, *t*_*r*_, **p**) are given by

$$a({{{\bf{p}}}},{t}_{i},{t}_{r})={a}_{{{{\rm{ion}}}}}{a}_{{{{\rm{prop}}}}}{a}_{{{{\rm{rec}}}}},$$

where

$${a}_{{{{\rm{ion}}}}}=\sqrt{\frac{2\uppi }{{\partial }_{{t}_{i}}^{2}S}}$$

$${a}_{{{{\rm{rec}}}}}=\sqrt{\frac{2\uppi }{{\partial }_{{t}_{r}}^{2}S}}$$

$${a}_{{{{\rm{prop}}}}}={\left(\frac{2\uppi }{{{{{i}}}}({t}_{r}-{t}_{i})}\right)}^{3/2};$$

the second derivatives of the action are given explicitly by

$${\partial }_{{t}_{i}}^{2}S=-{{{\bf{E}}}}({t}_{i})\cdot {{{\bf{k}}}}({t}_{i}),$$

$${\partial }_{{t}_{i}}^{2}S={{{\bf{E}}}}({t}_{r})\cdot {{{\bf{k}}}}({t}_{r}),$$

where **E**(*t*) is the electric field and all expressions for the prefactor are calculated at the complex times.

The transition matrix elements of the right- and left-handed molecules are related by

6 $${{{{\bf{D}}}}}_{\rm{R}}({{{\bf{k}}}})=-{{{{\bf{D}}}}}_{\rm{L}}(-{{{\bf{k}}}}),$$

whereas, for the overlap between the Dyson orbital and the Volkov wavefunction, we have

7 $${\Psi}_{D}^{\rm{R}}({{{\bf{k}}}})={\Psi}_{D}^{\rm{L}}(-{{{\bf{k}}}}).$$

The matrix elements and the Dyson orbitals for fenchone are calculated using DFT methods described in refs. ^47,48^.

### Multiphoton picture

The multiphoton picture of enantiosensitive HHG driven by chiral topological light can be understood by analysing the contributing chiral and achiral multiphoton pathways. To do so, we classify the multiphoton pathways by indicating with a subscript the SAM of the photon, so that, for example, (*N*)*ω*_+_ indicates the absorption of *Nω* photons with SAM *m* = 1, whereas (−1)*ω*_*z*_ indicates the emission of one *ω* photon with SAM *m* = 0.

In the specific case of bicircular counter-rotating fields, if the field has no longitudinal component along its direction of propagation (that is, if we consider an achiral field in the dipole approximation), conservation of SAM results in a harmonic spectrum with doublets at 3*N* + 1 and 3*N* + 2 harmonic frequencies, where the 3*N* + 1 harmonics (3*N* + 2) co-rotate with the *ω* (2*ω*) field^23,49^; 3*N* harmonic orders are forbidden in achiral media because their generation requires absorption of an equal number of photons from both drivers. In chiral media, the 3*N* harmonic orders can instead be generated due to the broken parity of the medium, but are polarized along the direction of propagation of the fields (the *z*-axis in our case), and thus are not detectable in the far-field. We label this pathway as

8 $${C}_{z}=\left[(N\,){\omega }_{+},(N\,)2{\omega }_{-}\right].$$

Focusing on the specific case of 3*N* harmonic orders, if the field is chiral (that is, if it possesses a longitudinal component along the propagation direction) in the case of achiral media, the following multiphoton pathways can now lead to symmetry-allowed HHG:

9 $${{{{\rm{AC}}}}}_{+}=\left[(N-2)\times {\omega }_{+},(2){\omega }_{z},(N-1)\times 2{\omega }_{-}\right]$$

10 $${{{{\rm{AC}}}}}_{-}=\left[(N-1)\times {\omega}_{+},(-1){\omega}_{z},(N\,)\times 2{\omega }_{-},(1)2{\omega }_{z}\right]$$

which correspond, respectively, to the emission of a photon with SAM *m* = 1 and *m* = −1. We label these pathways as achiral pathways (that is, AC_*m*_, with *m* representing the SAM of the harmonic photon) because they occur already in achiral media driven by a chiral field, and require the absorption and emission of an odd number of photons. If the medium is chiral, two new pathways including absorption of an equal number of *ω* and 2*ω* photons open, that is

11 $${{{{\rm{C}}}}}_{+}=\left[(N\,)\times {\omega }_{+},(N-1)\times 2{\omega }_{-},(1)2{\omega }_{z}\right]$$

12 $${{{{\rm{C}}}}}_{-}=\left[(N-1)\times {\omega }_{+},(1){\omega }_{z},(N\,)\times 2{\omega }_{-}\right]$$

corresponding again, respectively, to the emission of a photon with SAM *m* = 1 and *m* = −1. We label these pathways as chiral pathways (C_*m*_) because they can occur only in chiral media. Finding the corresponding OAM of all pathways indicated above is straightforward, once we remember that the longitudinal components of the fields carry OAMs of ${\ell }_{{\omega }_{z}}={\ell }_{{\omega }_{+}}+{\sigma }_{\omega }$ and ${\ell }_{2{\omega }_{z}}={\ell }_{2{\omega }_{-}}+{\sigma }_{2\omega }$. Obviously, other chiral and achiral pathways—including the absorption of a larger number of *z*-polarized photons from either drivers—are also in principle accessible; yet, as the longitudinal component is relatively weak, we restrict ourselves here to the photon pathways that include the absorption or emission of the fewest number of *z*-polarized photons. Supplementary Fig. 1a shows the multiphoton pathways C_*z*_, AC_*m*_ and C_*m*_ for a 3*N* harmonic order.

The results from the SFA simulations confirm the considerations above; in Supplementary Fig. 1b we show the near-field OAM distributions for H18 in *R*-fenchone driven by fields with *ℓ*_*ω*_ = −*ℓ*_2*ω*_ = 1 and *σ*_*ω*_ = − *σ*_2*ω*_ = 1. For comparison, we also report the OAM content for an artificial atom with an ionization potential that is equal to fenchone, driven by the same chiral field, and the OAM content in fenchone for an achiral field with driving beams with identical OAMs, which were obtained by manually setting the longitudinal component of the field to zero.

When the field is achiral, H18 is absent in an atom, whereas, in the case of fenchone we observe a *ℓ* = 0 component polarized along the *z*-axis: this corresponds to the pathway C_*z*_ denoted above. When the field is chiral, circularly polarized components with *ℓ* = ± 5 are observed for both the atom and the molecule: these are the achiral pathways AC_+_ and AC_−_ denoted above. Finally, the chiral pathways C_+_ and C_−_ correspond to the OAMs *ℓ* = ± 1 and are only seen in a chiral molecule because they require the absorption of an even number of photons. Note that only the SAM *m* = ± 1 components are going to be observed in the far-field because *m* = 0 polarization (corresponding to the C_*z*_ pathway in black in Supplementary Fig. 1b) will propagate in a direction orthogonal with respect to the propagation axis of the beams.

The difference in OAMs between an atom and chiral molecule driven by a chiral bicircular field is directly reflected in the far-field profile of H18 (Supplementary Fig. 1c). In an atom (left figure of Supplementary Fig. 1c), where there is only one contributing OAM for a given SAM, the far-field profile of H18 is a ring and the intensity is mostly constant, whereas, in fenchone, we observe an azimuthal interference pattern with periodicity determined by the topological charge *C*, which corresponds in modulus to the net difference between the OAMs of chiral and achiral pathways. The enantiosensitive rotation of the spatial profile can be understood from the perspective of the multiphoton pathways by accounting for a π shift of the phase of the chiral pathways *C*_±_ when changing the molecular enantiomer. The enantiosensitive rotation of the spatial profile of the high harmonics in the far-field allows one to also use HHG driven by chiral vortices as a highly sensitive method to infer the e.e. in a mixture of right- and left-handed molecular enantiomers.

Next-order pathways can be identified using the same approach. In the case of achiral channels, the next-order pathway includes the absorption of two more longitudinal photons (Supplementary Fig. 2), and is two orders of magnitude smaller. The next order-chiral pathway is four orders of magnitude smaller, corresponding to the absorption of four more longitudinal photons, and so on.

As mentioned in the main text in the case of an elliptically polarized *ω* field, two new achiral pathways dominate the response, whose photon diagrams we report in Supplementary Fig. 3. For a 3*N* harmonic order both new achiral pathways contribute to the final SAM of *m* = −1 and are in particular

13 $${{{{\rm{AC}}}}}_{1}^{\epsilon }=\left[(N-2)\times {\omega }_{+},(2){\omega }_{-},(N-1)\times 2{\omega }_{-}\right]$$

14 $${{{{\rm{AC}}}}}_{2}^{\epsilon }=\left[(N-1)\times {\omega }_{+},(-1){\omega }_{-},(N+1)\times 2{\omega }_{-}\right],$$

where *ω*_−_ refers now to the counter-rotating component of the elliptically polarized field at *ω* frequency. As each elliptically polarized photon carries a phase delay dependence of $\exp ({{{\rm{i}}}}\delta )$, the interference between these two achiral pathways oscillates with respect to the phase delay as 3*δ*. This explains why choosing the $\tilde{\delta }=1$ component of the harmonic profile after Fourier analysis allows one to recover the enantiosensitive rotation of the spatial profile.

### Noise (intensity fluctuations) simulations

To include the effect of noise on HHG driven by chiral vortex light, we take the following approach. For a given electric field strength *E*_0_ (which we assume to be the same for both fields), the Laguerre-Gaussian beam is given in the near-field by **E**(**r**) = *E*_0_**LG**_*l*,*p*_(**r**), where **LG**_*l*,*p*_ = LG_*l*,*p*_(**r**)**e**_*L*_(**r**). Here LG_*l*,*p*_ is a Laguerre-Gaussian mode and **e**_*L*_ is the polarization vector of the field. The corresponding laser intensity is *I*_0_ = ∣*E*_0_∣^2^. We then pick a value for the laser intensity from a normal distribution of noise centred at *I*_0_ with width *γ*. We call this electric field intensity *I*_1_. Then, for each point **r** in the focus, we introduce intensity fluctuations such that at a given position the electric field strength is given by

15 $${{{\mathcal{I}}}}({{{\bf{r}}}})={I}_{1}\,L{G}_{l,p}({{{\bf{r}}}})(1+{\delta }_{I}({{{\bf{r}}}})),$$

where *δ*_*I*_(**r**) = *Cλ*(**r**). *λ*(**r**) is chosen from a Gaussian distribution centred at zero with width 1 and *C* = 0.1 is a constant. There is therefore a 68.2% probability that the fluctuation is below 0.1% of the signal at the given point. We produce 16 electric fields using this approach, choosing a central intensity of *I*_0_ = 5 × 10^14^ W cm^–^^2^ with width *γ* = 3.51 × 10^13^ W cm^–^^2^, and calculate the resulting far-field picture for left- and right-handed fenchone. The average intensity fluctuations are on the order of 2%, on par with standard experimental parameters^50^. We then scan the e.e. between −100% and 100% in 1,001 steps. For each step, we pick a random index *i* between 1 and 16, selecting one of the far-field profiles for *R*- and *L*-fenchone ${{{{\bf{d}}}}}_{i}^{R/L}$. The resulting far-field image at an e.e. = (*N*_R_ − *N*_L_)/(*N*_R_ + *N*_L_) for normalized concentrations *N*_R_ + *N*_L_ = 1 is given by ${{{{\bf{d}}}}}^{ee}={N}_{R}{{{{\bf{d}}}}}_{i}^{R}+{N}_{L}{{{{\bf{d}}}}}_{i}^{L}$ and the phase of the *ℓ* = 6 Fourier component of the outer ring *kW*_0_ > 10 is then calculated. We then repeat the procedure 16 times and for each e.e. calculate the mean phase as $\bar{\phi }=\mathop{\sum }_{i = 1}^{16}{\phi }_{i}/16$. The result is the red solid line shown in Fig. 3e.

## Online content

Any methods, additional references, Nature Portfolio reporting summaries, source data, extended data, supplementary information, acknowledgements, peer review information; details of author contributions and competing interests; and statements of data and code availability are available at 10.1038/s41566-024-01499-8.

## Supplementary information

Supplementary Information Supplementary Figs. 1–5 and discussion.

## Data Availability

The data used to generate the figures resulting from the numerical simulations in this paper have been publicly archived in the Zenodo repository at 10.5281/zenodo.11501346 (ref. ^51^).

## Code availability

The code used to generate the data is available from the authors on reasonable request.
