# Supplementary material for: Chiral topological light for detection of robust enantiosensitive observables
Source: Nat Photonics. 2024 Aug 13;18(11):1155–60. doi: 10.1038/s41566-024-01499-8 (PMC11527792; doi:10.1038/s41566-024-01499-8)
Supplement: Supplementary file 1 — Supplementary Figs. 1–5 and discussion. [file 41566_2024_1499_MOESM1_ESM.pdf]

nature photonics

Supplementary information

https://doi.org/10.1038/s41566-024-01499-8

Chiral topological light for detection of robust enantiosensitive observables

In the format provided by the authors and unedited

Contents

1 Multiphoton analysis **2**

2 Results for beams including imperfect OAM **2**

3 Proposal for an experimental setup **7**

1 Multiphoton analysis

Fig. 1 shows the multiphoton picture of CTL driven HHG in the case of perfect circularly polarized fields carrying OAM $\ell_{r\omega}$. This includes the relevant pathways (Fig. 1a), the near-field distribution of OAM for different SAM components Fig. 2 in the case of an (achiral) atom driven by CTL, a chiral molecule driven by an achiral bicircular $\omega - 2\omega$ field and a chiral molecule driven by a chiral bicircular field with topological charge $C = 6$. Fig. 1c shows the corresponding far-field spatial profiles. Fig. 2 shows the next leading order achiral (AC) and chiral (C) pathways. Fig. 3 shows the elliptically polarized driven achiral pathways AC_1^ϵ and AC_2^ϵ mentioned in the Methods section.

2 Results for beams including imperfect OAM

We consider the fundamental ω RCP field to carry OAM that are different than $\ell = 1$ as in the main text, i.e.

$$\mathbf{E}(\omega) = \sum_{\ell} c_\ell \mathbf{E}(\omega)_\ell \tag{1}$$

where we normalize the field such that its peak total intensity is the same as in the purely $\ell = 1$ case. In particular we did simulations for a field carrying a 10% component at $\ell = 0$ or $\ell = 2$ with respect to the main $\ell = 1$ one, as well as a simulation where the field has both $\ell = 0$ and $\ell = 2$ components, both at 10% of the main $\ell = 1$ one. In all simulations we assume that the LCP $\mathbf{E}(2\omega)$ field has only $\ell = -1$ OAM. The results are summarized in Fig. 4. For each simulation we show the near-field intensity of the $\mathbf{E}(\omega)$ field, the total intensity of the two-color field $\mathbf{E}(\omega) + \mathbf{E}(2\omega)$, the absolute value of the chiral correlation function $|h^{(5)}|$ and its phase $\arg[h^{(5)}]$. It is clear that while the topological charge $C = 6$ of the chiral beam is mostly not affected by the presence of additional ℓ_ω components, its absolute value (i.e. the degree of chirality) reflects the azimuthal shift of the intensity of the ω beam, as one would expect. The resulting near- and far-field profiles for the molecular enantiomers, as well as their difference $S_R - S_L$ in the far-field, are also reported. For both the case of either 0.1% additional content of $\ell = 0$ or $\ell = 2$ in the ω beam the enatiosensitive rotation is still clearly present and is simply shifted off-axis with respect to the beam propagation axis. In the case of a 0.1% of both $\ell = 0$ and $\ell = 2$, the intensity gating of the $h^{(5)}$ chiral correlation function is so strong that the six-fold interference in the near-field is partly lost, leading to a far-field profile that loses the six-fold structure. Yet the difference between the far-field profiles of opposite molecular enantiomers is still very much apparent. The intensity gating due to additional OAM content could easily be adjusted by either changing the topological charge of the beam in order to still retain most of the azimuthal interferences.

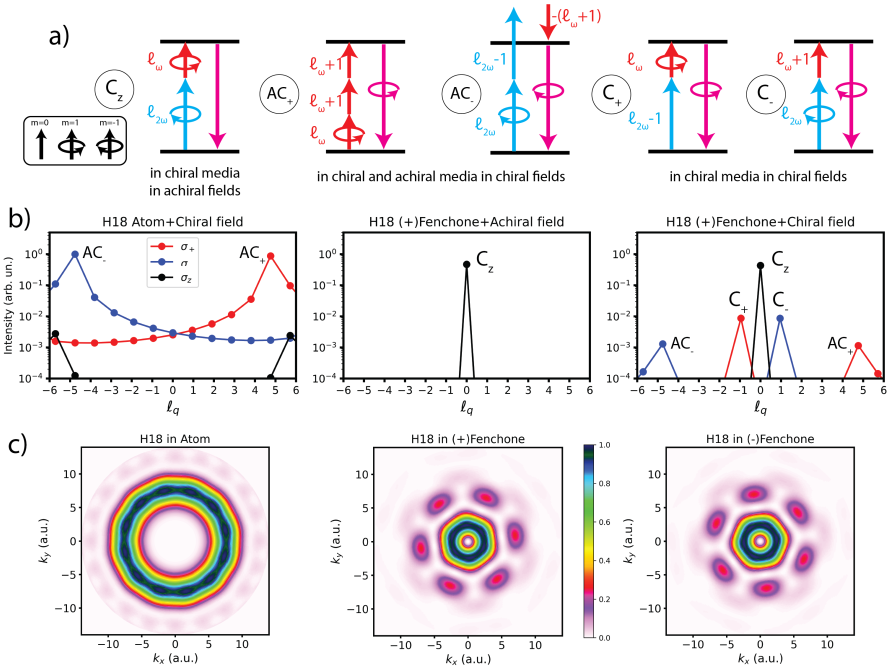

Figure 1: OAM analysis of HHG driven by chiral topological light. **a)** Pathways to 3N harmonics in bicircular fields carrying OAM. The pathways are classified according to the final SAM of the harmonic photon ($m = -1, 0, 1$ corresponding respectively to the subscripts $-, z, +$) and their occurrence in achiral (AC) or chiral (C) media. **b)** Near-field OAM distributions for H18 in a bicircular field with $\ell_\omega = -\ell_{2\omega} = 1$. The left figure shows the OAM content of H18 for an atom in the presence of a chiral field: only the pathways $AC_\pm$ are allowed; the middle figure shows the OAM content for the chiral molecule (+)Fenchone in the presence of an achiral field: only the pathway C_z is allowed; the right figure shows the OAM content for (+)Fenchone in the presence of a chiral field: all pathways are allowed. **c)** Far-field H18 spatial profiles for an atom (left), (+)Fenchone (middle) and (-)Fenchone (right) driven by a chiral vortex with $\ell_\omega = -\ell_{2\omega} = 1$ and $C = -6$.

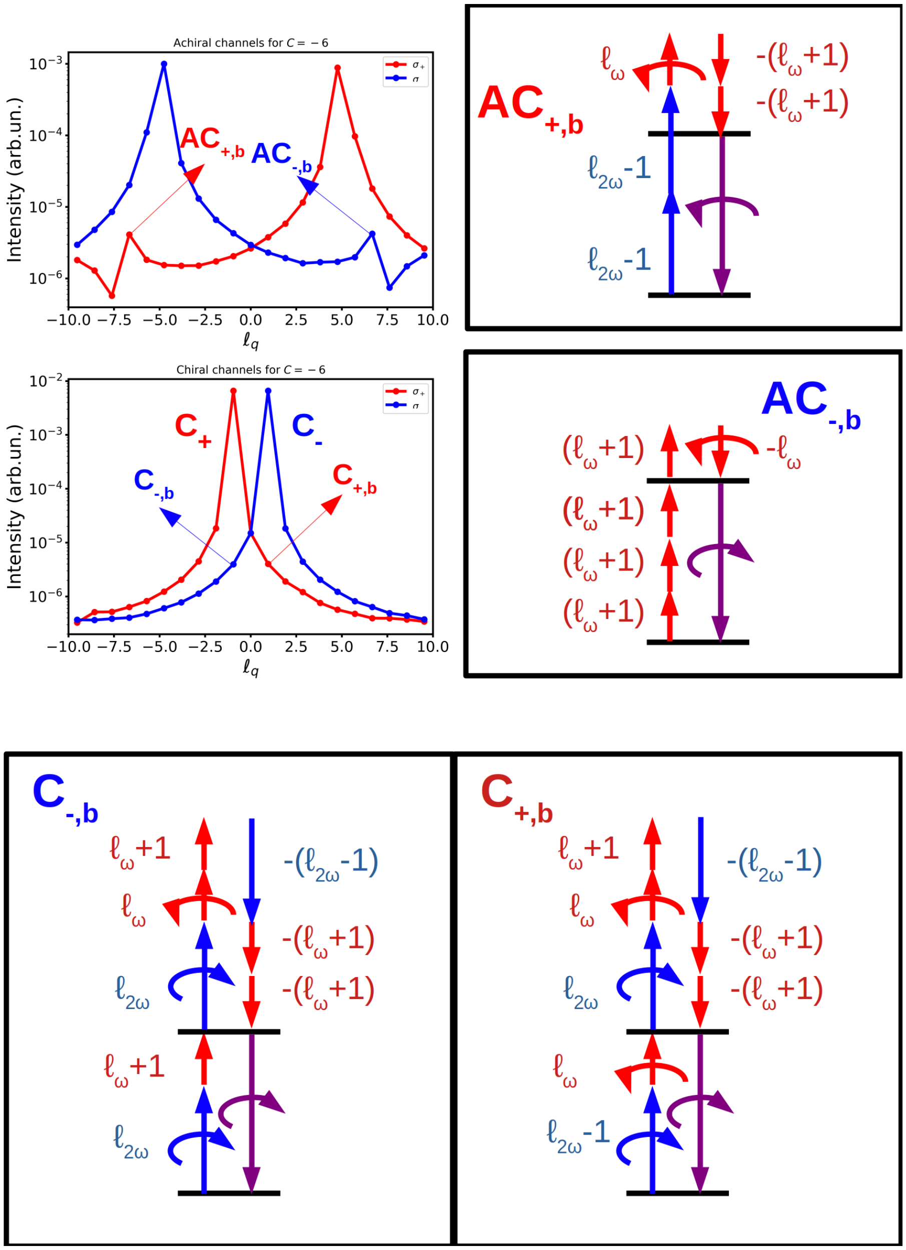

Figure 2: OAM analysis of achiral and chiral pathways to H18 for a field with topological charge $C = -6$. The pathways $C_\pm$ and $AC_\pm$ correspond to the ones of the main text. In the figure we also show the next-order contributions to achiral channels $AC_{\pm,b}$, as well as the next-order contributions to the chiral channels $C_{\pm,b}$, together with the corresponding diagrams. Next-order contributions to achiral channels $AC_{\pm,b}$ include the absorption and emission of two additional longitudinally polarized photons, and they are correspondingly two orders of magnitude smaller than the dominant $AC_\pm$ ones. The next-order contributions to chiral channels $C_{\pm,b}$ include instead the absorption and emission of four more longitudinally polarized photons, and their contribution is negligible with respect to the dominant $C_\pm$ channels. We indicate their predicted OAM content for reference.

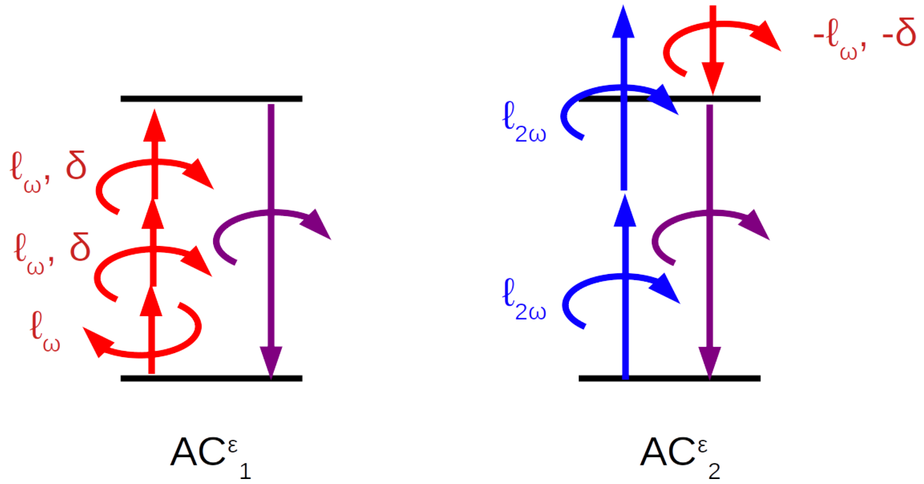

Figure 3: New achiral pathways driven by elliptically polarized ω fields. δ refers to the dependence of the weak counter-rotating component at ω frequency with respect to the phase delay δ between the CP components of the elliptically polarized field. For $\ell_\omega = 1$, $\ell_{2\omega} = -1$ the AC_1^ϵ pathway leads to a final 3N harmonic with SAM $m = -1$ having $\ell = 3$ and a dependence on the phase delay of $\exp(\mathrm{i}2\delta)$, while for AC_2^ϵ we have $\ell = -3$ and $\exp(-\mathrm{i}\delta)$, leading to an interference pattern with $\ell = 6$ and a phase delay dependence of 3δ.

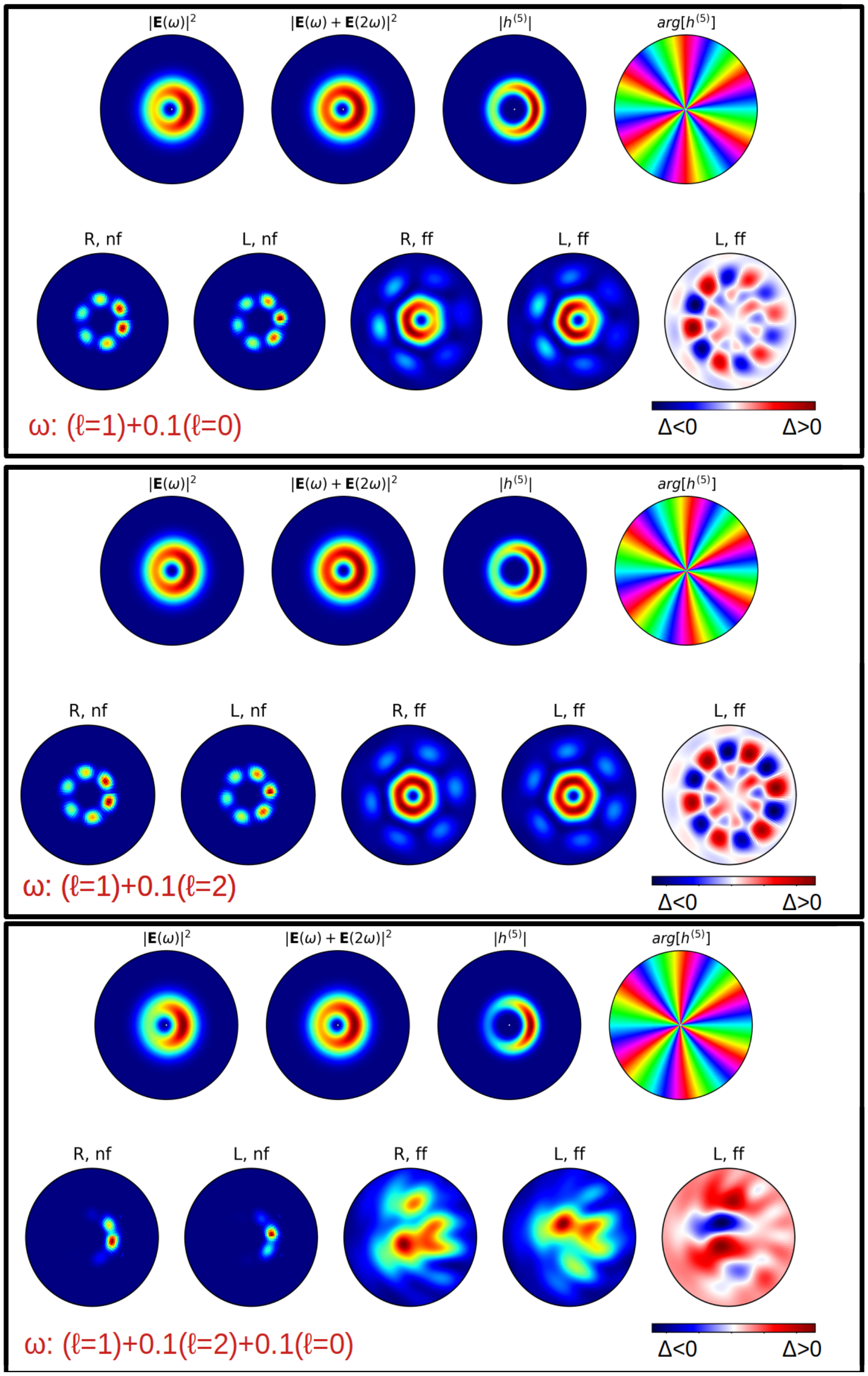

Figure 4: Results for a RCP ω beam carrying additional OAMs at 0.1% of the predominant $\ell = 1$ one in combination with a LCP 2ω beam carrying $\ell_{2\omega} = -1$.

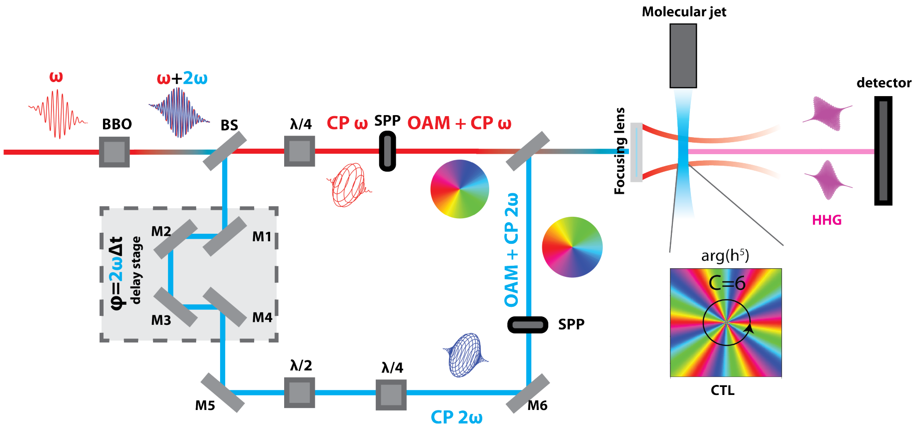

Figure 5: Experimental setup for enantiosensitive high-harmonic spectroscopy using chiral topological light. An incoming intense linearly polarized beam at ω frequency passes through a BBO (beta barium borate), generating a 2ω linearly polarized field. The two colors are then split by a beamsplitter (BS). After a delay stage in the 2ω arm that introduces a controlled relative phase between the two frequencies, the two linearly polarized fields pass through a combination of $\lambda/2$ and $\lambda/4$ polarizers to obtain counter-rotating circularly polarized fields ($\epsilon = 95\%$ ellipticity is well within reach, see e.g. Ref. [1]). They then pass through spiral-phase plates (SPP) which introduce orbital angular momentum in the beams (see e.g. [2]). They are then recollimated on a focusing lens, which tightly focus the two color field to obtain chiral topological light with a chiral topological charge C. A thin gas jet of chiral molecules is placed before the laser focus, as in standard HHG experiments, and enantiosensitive high-harmonic radiation is emitted and detected in the far-field.

3 Proposal for an experimental setup

Here we sketch a possible experimental setup aimed at detecting enantiosensitive high-harmonic generation in chiral molecules driven by chiral topological light, based on existing table-top experimental setups [1]. The setup and its description are given in Fig. 5.

References

[1] Álvaro Jiménez-Galán, Nickolai Zhavoronkov, Marcel Schloz, Felipe Morales, and Misha Ivanov. Time-resolved high harmonic spectroscopy of dynamical symmetry breaking in bi-circular laser fields: the role of rydberg states. *Optics express*, 25(19):22880–22896, 2017.

[2] K. M. Dorney, L. Rego, N.J. Brooks, and et al. Controlling the polarization and vortex charge of attosecond high-harmonic beams via simultaneous spin–orbit momentum conservation. *Nat. Photon.*, 13:123–130, 2019.
